# Adsorption of Sb(III) from Aqueous Solution by nZVI/AC: A Magnetic Fixed-Bed Column Study

**DOI:** 10.3390/nano11081912

**Published:** 2021-07-25

**Authors:** Huijie Zhu, Qiang Huang, Mingyan Shi, Shuai Fu, Xiuji Zhang, Zhe Yang, Jianhong Lu, Bo Liu

**Affiliations:** 1 Henan International Joint Laboratory of New Civil Engineering Structure, College of Civil Engineering, Luoyang Institute of Science and Technology, Luoyang 471023, China; huijiezhu@lit.edu.cn (H.Z.); shuaifu16@163.com (S.F.); zhangxiuji@lit.edu.cn (X.Z.); kaiyzhey17947@126.com (Z.Y.); 2 College of Civil Engineering, Guangzhou University, Guangzhou 510006, China; shmygz@gzhu.edu.cn; 3 School of Environmental and Municipal Engineering, North China University of Water Resources and Electric Power (NCWU), Zhengzhou 450000, China; lujianhong@ncwu.edu.cn; 4 Laboratory of Functional Molecular and Materials, School of Physics and Optoelectronic Engineering, Shandong University of Technology, Zibo 255000, China

**Keywords:** fixed-bed column, antimonite (Sb(III)), weak magnetic field (WMF), adsorption, removal

## Abstract

The effectiveness of nanoscale zero-valent iron(nZVI) immobilized on activated carbon (nZVI/AC) in removing antimonite (Sb(III)) from simulated contaminated water was investigated with and without a magnetic fix-bed column reactor. The experiments were all conducted in fixed-bed columns. A weak magnetic field (WMF) was proposed to increase the exclusion of paramagnetic Sb(III) ions by nZVI/AC. The Sb(III) adsorption to the nZVI and AC surfaces, as well as the transformation of Sb(III) to Sb(V) by them, were both increased by using a WMF in nZVI/AC. The increased sequestration of Sb(III) by nZVI/AC in the presence of WMF was followed by faster nZVI corrosion and dissolution. Experiments were conducted as a function of the pH of the feed solution (pH 5.0–9.0), liquid flow rate (5–15 mL·min^−1^), starting Sb(III) concentration (0.5–1.5 mg·L^−1^), bed height nZVI/AC (10–40 cm), and starting Sb(III) concentration (0.5–1.5 mg·L^−1^). By analyzing the breakthrough curves generated by different flow rates, different pH values, different inlet Sb(III) concentrations, and different bed heights, the adsorbed amounts, equilibrium nZVI uptakes, and total Sb(III) removal percentage were calculated in relation to effluent volumes. At pH 5.0, the longest nZVI breakthrough time and maximal Sb(III) adsorption were achieved. The findings revealed that the column performed effectively at the lowest flow rate. With increasing bed height, column bed capacity and exhaustion time increased as well. Increasing the Sb(III) initial concentration from 0.5 to 1.5 mg·L^−1^ resulted in the rise of adsorption bed capacity from 3.45 to 6.33 mg·g^−1^.

## 1. Introduction

Antimony (Sb) is the ninth-largest mined metal and is used in a variety of industrial products [1]. China produces over 88% of the world’s commercial antimony, and large quantities of antimony are discharged into the environment as a result of Sb smelting and mining processes, resulting in major contamination of soil and water. Concentrations of antimony up to 7.3–163.0 μg·L^−1^ have been found in natural waterways from the world’s largest Sb mine, Xikuangshan in Hunan Province, China [2].

Long-term exposure to high-level Sb-contaminated water has negative consequences that can lead to serious health problems. Many governments and regions have adopted measures to regulate the amount of Sb in aqueous solutions. The most common environmental antimony species are Sb(III) and Sb(V), of which Sb(III) is 10 times more hazardous than Sb(V) [1]. The World Health Organization (WHO) and China have issued a standard for Sb in drinking water at 0.005 mg·L^−1^.

Ion exchange [3], coagulation (coprecipitation) [4], reverse osmosis [5], bioremediation [6], and adsorption [7] are currently accessible technologies for Sb(III) removal from water. Adsorption is a common approach for removing Sb(III) from drinking water due to its economic and technological advantages. Iron (hydr)oxides and elemental iron are commonly used as antimony adsorbents due to their high affinity for Sb(III) and Sb(V) [8]. In developing countries, zero-valent iron powders are utilized in permeable reactive barriers to intercept Sb(III) plumes in contaminated groundwater or added to household filters to remove Sb(III) [1]. Nanoscale zero-valent iron (nZVI) has recently been identified as a promising material for in situ remediations of antimony contaminated water [9,10,11,12,13,14]. Due to its high Sb(III) adsorption capability and huge active surface area, it can serve as a viable material to remove Sb(III) from drinking water. Iron (hydr)oxides and Zero-valent iron, on the other hand, are usually fine powders that cannot be used directly in fixed-bed columns until they are converted into granular form [10]. Ferric hydroxide can be handled as a granulated material or applied to porous carbon, sand, or other materials for fixed-bed water treatment operations. Due to its smaller particle size, direct use of nZVI in water treatment systems may result in rapid nZVI loss and iron pollution in water. Therefore, to treat Sb(III)-contaminated drinking water, nZVI must be loaded onto supporting materials. The batch technique has received greater attention for Sb(III) adsorption. However, adsorption techniques based on the removal of heavy metals carried out in column systems is regarded to be particularly advantageous from an industrial standpoint, due to the column systems’ adaptability to diverse procedures, reduced reagent handling, and consequently low functioning costs [10,11,12,13,14]. As a result, adsorption investigations utilizing columns are required.

Breakthrough experiments are being conducted to assess performance in continuous, fixed-bed operations by altering operating variables such as bed depth, flow rate, influent Sb(III) pH, and concentration. The breakthrough curve for Sb adsorption was studied using the Thomas and BDST models.

There have been several batch studies on nZVI or its derivatives-based removal of heavy metals, but only a few experiments on the packed bed column system have been documented. The fixed-bed column can mimic as closely as possible the actual operating conditions of wastewater treatment.

It has recently been revealed that employing a weak magnetic field (WMF) to boost ZVI performance for sequestering various contaminants is an effective and low-cost environmentally friendly strategy that can accelerate ZVI corrosion. The ZVI/WMF approach has been shown to effectively remove Sb(III)/Sb(V), Se(IV)/Se(VI) [7], As(III)/As(V) [8], Cr(VI) [15], Cu(II), and Ni [16]. Xu et al. [17] found that adding WMF to the process improved the ZVI-based elimination of Sb(III)-tartrate, but they did not discuss the oxidation–reduction behavior of Sb(III) or the impact of tartrate on the removal behavior of Sb(III). Furthermore, batch experiments were used to obtain the majority of published data on the ZVI/WMF methodology. Thus, investigation is required into the effect of WMF on removing potential ZVI contaminants in continuous or semi-continuous tests to encourage the use of the ZVI/WMF approach.

This study’s goal is to look into the performance of nZVI immobilized on AC (nZVI/AC) in removing Sb(III) from aqueous solutions in a fixed-bed system. Due to its admirable properties in porous structures and mechanical strength, activated carbon can be used as a supporting material (though its adsorption capacity for Sb(III) is much lower than that of nZVI). The effects of various parameters, i.e., adsorbent dosage, initial concentration, pH, flow rate, and the shape of the Sb(III) removal breakthrough curves were also examined.

## 2. Materials and Methods

### 2.1. Preparation and Characterization of nZVI/AC

nZVI/AC was synthesized by soaking the carbon in HNO_3_ (Sinopharm Chemical Reagent Co., Ltd., Shanghai, China ) for washing then rinsing it several times with distilled water. AC was equilibrated with a solution of ferrous sulfate. The solution was then diluted with an aqueous solution of ethanol. In the presence of N_2_ bubbling and magnetic stirring, ferrous iron (Fe^2+^) was reduced by the addition of NaBH_4_. After agitation, the nZVI supported by the AC was separated and washed with acetone before being vacuum dried and stored in an N_2_-purged drying chamber [8,13]. Table 1 summarizes its key characteristics.

### 2.2. Chemical and Instrumentation

All of the compounds were of analytical grade, and all solutions were prepared with DI water. Sb_2_O_3_ (Sinopharm Chemical Reagent Co., Ltd., Shanghai, China ) was dissolved in 2.0 mol·L^−1^ HCl (Sinopharm Chemical Reagent Co., Ltd., Shanghai, China ) to make 100 mg·L^−1^ stock solutions Sb(III).

Sb(III) was measured using an atomic fluorescence photometer (AFS-8220, Beijing Jitian Instrument Co., Ltd., Beijing, China). In the fixed-bed column, a peristaltic pump (BT-100, Shanghai Huxi Instruments Company, Shanghai, China) was also utilized to provide a steady flow of desorbing solution and metal ions.

### 2.3. Fixed Bed Column Study

**Figure 1:**
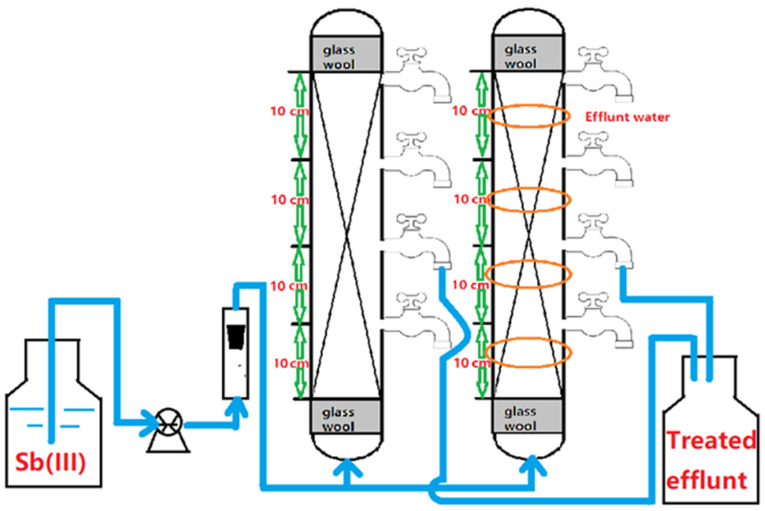
Schematic representation of the setup for the continuous flow system tests.

We carried out the column experiments to see if WMF could be used to improve the reactivity of nZVI in a continuous flow system. Two parallel continuous column systems with 10 mm of inner diameter (outer diameter = 14 mm) and a height of 200 mm were set up, as shown in Figure 1. One column system had a series of ring-shaped permanent magnets with a maximum magnetic field flux intensity of ~1800 Gs (Material: NdFeB, inner diameter = 14 mm, outer diameter = 25 mm, thickness = 5 mm, Shenzhen Min Magnetic Technology Co., Ltd., Shenzhen, China), which placed a ring magnet every 5.0 cm [18]. The polymethyl methacrylate (PMMA) columns were filled with a mixture of 10.62 g of nZVI/AC (10–20 mesh). Both ends of the column were plugged with a little piece of glass wool. Figure 1 depicts the column component and experimental design that go with it. The tests were accomplished at 25 ± 1 °C. To achieve an appropriate flow rate, a peristaltic pump (HL-1S, Shanghai Huxi Analytical Instrument Co., Shanghai, China) was utilized. The Sb(III) ion solution was transferred upward via the nZVI/AC beds to ensure perfect saturation of the bed. To assess the conservation of Sb(III), effluent samples were taken at regular intervals.

The exhausted nZVI/AC was removed from the column and kept in a 250 mL flask for regeneration. After shaking the suspension for 5 h at 150 rpm at 25 ± 1 °C with a NaOH (Sinopharm Chemical Reagent Co., Ltd., Shanghai, China ) solution (0.5 mol·L^−1^), which was 5 times the nZVI/AC volume, the alkaline solution was discarded. The desorption process was repeated four times with deionized water.

### 2.4. Equilibrium Uptake Studies

For a given flow rate and inlet concentration, the q_total_ (mg) (maximum column capacity), is equal to the area under the plot of the concentration of adsorbed Sb(III) C_ad_ (C_ad_ = C_0_ − C_e_, where C_e_ is the concentration of effluent metal ions and C_0_ is the concentration of influent metal ions) (mg·L^−1^) versus time (h) and can be calculated as [15]:

(1) $$q_{\mathrm{total}}=\frac{\mathrm{QA}}{1000}=\frac{Q}{1000}\int_{t=0}^{{t=t}_{\mathrm{total}}}C_{\mathrm{ad}}\mathrm{dt}$$

(2) $$q_{\mathrm{eq}(\exp)}=\frac{q_{\mathrm{total}}}{X}\;(2)$$

where X represents the total dry weight of nZVI/AC in column (g).

### 2.5. Analytical Methods

The suspension (5 mL) was taken at specified time intervals for column experiments, filtered by using a membrane filter (Shanghai xinya purification equipment Co., Ltd., Shanghai, China) of 0.22 μm, and acidified for examination. The concentration of Sb(III) was determined using colorimetric techniques and an atomic fluorescence photometer (AFS–8220, Beijing Jitian Instrument Co., Ltd., Beijing, China) [19]. ICP-MS was used to evaluate the total concentration of dissolved Fe in the effluent of the nZVI/AC-packed columns.

## 3. Results and Discussion

### 3.1. Effect of Bed Height

**Figure 2:**
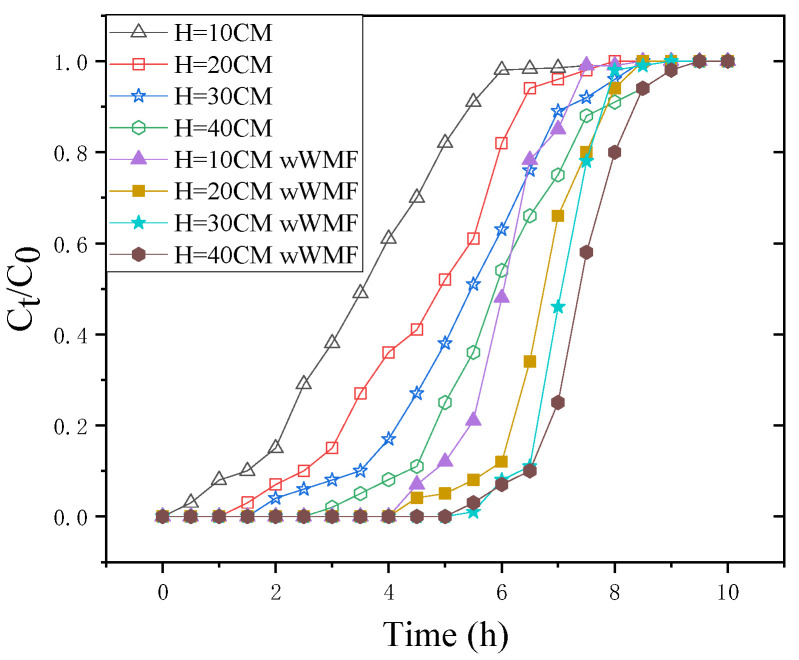
Breakthrough curves of Sb(III) removal by nZNI/AC for different bed depths (wWMF = with weak magnetic field). H = 10, 20, 30, and 40 cm; C_0_ = 1 mg·L^−1^; flow rate = 10 mL·min^−1^; pH = 7.

The amount of sorbent inside the packed bed column determines how much Sb(III) accumulates in the column. nZNI/AC amounts of 5.31, 10.62, 15.93, and 21.24 g were used to achieve bed heights of 10, 20, 30, and 40 cm, respectively. Figure 2 shows the sorption breakthrough curves for nZNI/AC produced by adjusting the bed heights of 10 to 40 cm with 1 mg·L^−1^ initial Sb(III) concentration at 10 mL·min^−1^ flow rate. Adsorption increased with bed depth due to higher adsorbent dosages in larger beds, providing more fixing binding sites for Sb (III). Following that, a delayed pollutant breakthrough resulted in an increased treated solution volume. The adsorption enhancement with increasing bed depth was attributed to higher absorbent doses in larger beds with more adsorption sites. With the lowered bed depth, axial dispersion processes took over in mass transfer, reducing Sb(III) ion diffusion. The Sb(III) ions were unable to diffuse across the entire bulk of the adsorbent due to a lack of time.

As predicted, increasing the bed size led to a higher volume of treated Sb(III) solution and a higher proportion of Sb(III) omission. The total Sb(III) uptake increased from 1.42 to 2.425 to 3.385 to 4.397 g while increasing the bed height from 10 to 20 to 30 to 40 cm, respectively. However, the equilibrium uptake (qe) decreased from 0.1605 to 0.1370 to 0.1275 to 0.1242 mg·g^−1^ when increasing the bed height from 10 to 20 to 30 to 40 cm, respectively. Over the same range of bed heights, the total Sb(III) uptake with WMF increased from 3.474 to 2.427 to 1.899 to 1.674 times, respectively. Unlike the sequencing batch experiment that increases the removal effect of antimony with a weak magnetic field, the dissolved iron is also lost from the fixed bed, resulting in a poorer antimony removal effect [7].

### 3.2. Influence of Flow Rate

**Figure 3:**
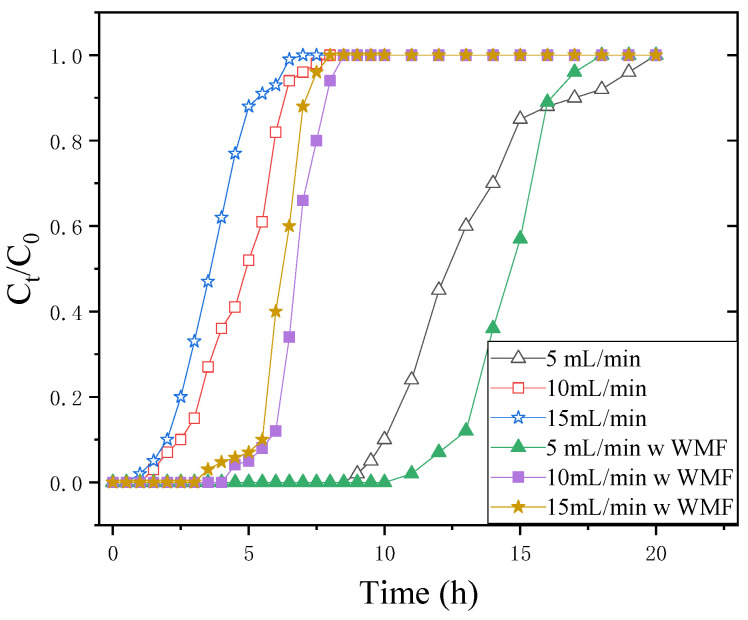
Breakthrough curves for nZNI/AC mediated Sb(III) removal at various flow rates. H = 20 cm; C_0_ = 1 mg·L^−1^; pH = 7; flow rate = 5, 10, and 15 mL·min^−1^.

By adjusting the flow rate from 5 to 15 m·min^−^^1^ with constant initial Sb(III) concentration (1.0 mg·L^−^^1^) and bed depth (20 cm), flow rate impact on Sb(III) adsorption by nZNI/AC was examined. Figure 3 shows a plot of normalized Sb(III) concentration vs. time at several flow rates.

The presence of reaction sites capable of capturing Sb(III) around or inside the adsorbents caused the adsorption to be highly rapid at low flow rates at first. Due to the gradual occupancy of these areas, the less effective uptakes were observed in the subsequent step of the process. Even after breaking through, the column can accumulate Sb(III). Increased flow rate resulted in an increase in the steepness of the breakthrough curve, reducing the breakpoint time and concentration of adsorbed Sb(III). The most likely explanation is that the Sb(III) solution leaves the column before it is equilibrated due to inadequate solute residence time in the column to establish adsorption equilibrium at that flow rate. As a result, at higher flow rates, the contact period of Sb(III) with nZNI/AC is relatively brief, resulting in a decrease in absorption capacity [12,14].

By increasing the flow rate to 15 mL·min^−1^, there was an exhaustion time and earlier breakthrough in the profile. When further increased to 10 mL·min^−1^, the capacity for Sb(III) uptake decreased from 0.2808 to 0.1370 mg·g^−1^. When increasing the flow rate, the volume of effluent treated up to breakthrough concentration decreased, with the greatest volume of 5.85 L being reached at 15 mL·min^−1^. At tested flow rates of 5, 10, and 15 mL·min^−1^, the mass of uptake Sb(III) was 9.94, 2.425, and 1.94 mg·g^−1^, respectively, and the q_eq_ of nZNI/AC were 0.2808, 0.1370, and 0.1644 mg·g^−1^, respectively without WMF.

When the flow rate is 5, 10, and 15 mL·min^−1^, the dynamic adsorption penetration curves of Sb(III) are, respectively 129.3%, 243.0%, and 276.9% when there is no WMF. nZVI accelerates corrosion by WMF, and the produced Fe^2+^ promoted the removal of Sb(III).

### 3.3. Influence of Initial Concentration

**Figure 4:**
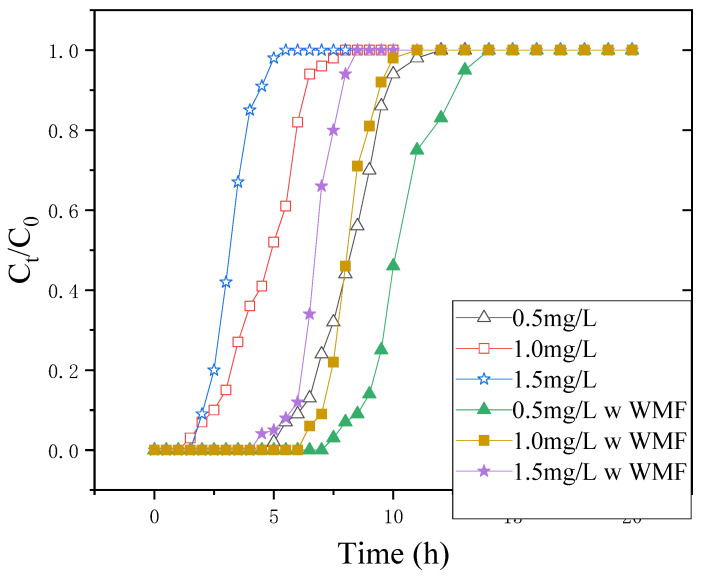
Breakthrough curves of Sb(III) removal by nZNI/AC for different concentrations. Flow rate = 10 mL·min^−1^; H = 20 cm; pH = 7; C_0_ = 0.5, 1, 2 mg·L^−1^.

A synthetic solution comprising 0.5, 1.0, and 1.5 mg·L^−1^ of Sb(III) ions was used to observe the influence of the initial concentration of influent. Using a 20 cm bed depth and 10 mL·min^−1^ flow rate, we presented the influence of initial concentration on the breakthrough curves in Figure 4. A rise in the incoming concentration of metal decreased the treated volume before saturation of the fixed adsorption bed; however, a high metal content quickly saturates the absorbents, reducing service periods (ta). The q_eq(exp)_ of nZNI/AC at 0.5, 1.0, and 1.5 mg·L^−1^ Sb(III) ions concentrations were 3.54, 5.497, and 6.33 mg·g^−1^, respectively, for testing different initial concentrations.

By lowering the concentration of Sb(III) ions in the feed, the treated volume of feed metal concentration was increased with the pushing of breakthrough curves to the right. The concentration changes between the solute present on the absorbent and in solution proceeded to drive adsorption. A large difference in the concentration generated a strong driving force for the process of adsorption, which could explain why the column fed with more Sb(III) ions had higher adsorption capacities. The variation in surface morphology could explain the relatively low Sb(III) ion retention for adsorbents.

At different parameters, the ta and q_eq(exp)_ of nZNI/AC were higher and longer when compared to that of WMF.

Figure 4 depicts the influence of WMF on the nZNI/AC-based elimination of Sb(III) at various initial Sb(III) concentrations and Fe^2+^ release during this process at pH 7.0. The rate of Sb(III) removal by nZNI/AC was dramatically reduced with decreasing initial Sb(III) in the absence of WMF over the range of starting Sb(III) concentrations studied. In the absence of WMF, a higher Sb(III) clearance rate was accompanied by a more notable Fe^2+^ release.

Furthermore, the WMF presence increased the release of Fe^2+^ significantly. Regardless of the initial concentration of Sb(III), the quicker elimination of Sb(III) due to the presence of WMF was accompanied by a higher release of Fe^2+^. Figure 2 also shows the significant impact of WMF on Fe^2+^ release at the lowest Sb(III) concentration of 0.5 mg·L^−1^, which corresponds to its impact on Sb(III) elimination. According to the observations at various pH levels, WMF’s significant influence on Sb(III) elimination was directly related to its effect on Fe^2+^ accumulation. When Liang removed selenite with zero-valent iron and a modest magnetic field, he got identical results [16].

### 3.4. Influence of pH

The adsorption medium pH is the single most critical factor regulating sorption capacity. The nature of the interaction (physicochemical) between the species in adsorptive sites of the adsorbent and solution is reflected in the initial pH of the adsorption medium, which is connected to the mechanism of adsorption onto the surface of adsorbent from water. The influence of pH on breakthrough time was investigated at different pH levels (pH 5 to pH 9) while maintaining the same flow rate, bed height, and initial concentration.

**Figure 5:**
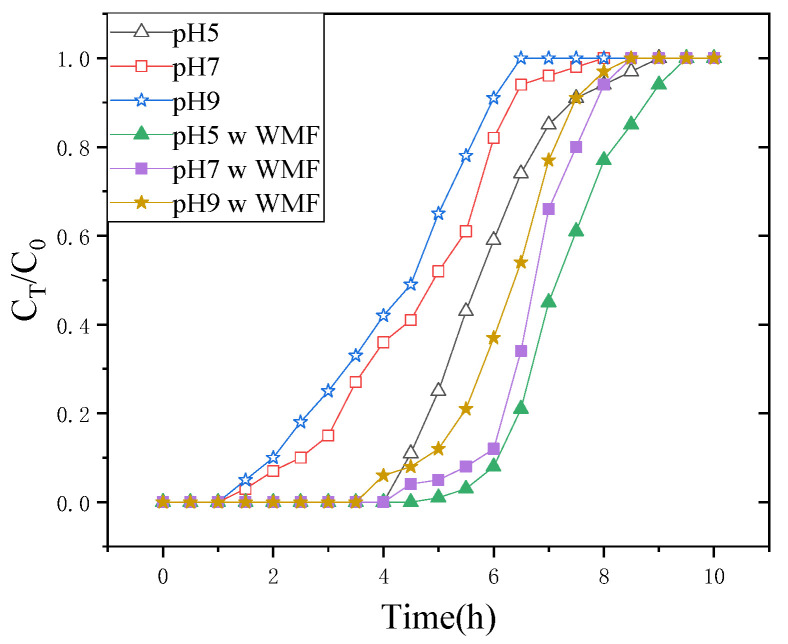
Breakthrough curves of nZNI/AC mediated removal of Sb(III) for different pH. pH = 5, 7, and 9; flow rate = 10 mL·min^−1^; H = 20 cm; C_0_ = 1 mg·L^−1^.

The plot of normalized concentration (C_t_/C_0_) versus time in Figure 5 shows the effect of pH values on Sb(III) adsorption onto nZNI/AC. When the pH was 5.0, the C_t_/C_0_ value extended to 0.95 in 8 h, as illustrated in Figure 5. However, for the pH 7 and 9 breakthrough curves, this value was 7 h and 6 h, respectively. The breakthrough curves shifted from right to left as the pH of the influent increased, indicating that less Sb(III) could be removed, that it would take a shorter time to reach saturation, and that the sorption efficiency would be substantially lower. The findings indicated that as the pH in the experimental environment rises, the adsorption capabilities decrease. As a result, at lower initial pH values, the exclusion of Sb(III) was more efficient from the aqueous solution. The acidic medium can also promote the release ability of Fe^2+^ from nZVI/AC, resulting in the formation of a large amount of iron oxides. Therefore, nZVI/AC exhibits the highest removal efficiency of Sb(III) at pH 5. By contrast, the electrostatic adsorption capacity and release ability of Fe^2+^ in nZVI/AC is weak in alkaline conditions, thus the removal ability of Sb(III) is relatively low. Liu found the removal capacity of Sb(III) by S-nZVI slightly reduces with the increase in solution pH [16].

The total quantities of adsorbed Sb(III) and adsorbent uptake capacity decreased from 0.1605 to 0.1370 to 0.1275 mg·g^−1^ (nZNI/AC) at pH 5, 7, and 9, respectively.

WMF has an effect on Sb(III) sequestration using nZNI/AC at various pH, as shown in Figure 5. At the pH range of 5.0–9.0, the WMF caused a substantial increase in the mass of removed Sb(III). When the pH was raised from 5.0 to 9.0, the amount of Sb(III) adsorbed and the adsorbent’s absorption capacity rose by 1.7139, 1.4544, and 1.3133 times at pH 5, 7, and 9, respectively. Sb(III) removal by nZNI/AC is accompanied by the release of Fe^2+^. In the absence of WMF, the Sb(III) release rate decreased sharply with increasing pH, attributing to the sharp decline in Sb(III) removal rate with increasing pH. The use of WMF significantly increased the rate of Fe^2+^ release, which accelerated the removal of Sb (III).

There is a lag phase before the onset of a period of quick removal for Sb(III) at pH 5.0–9.0 in the absence of WMF. The initial period of the lag phase was thought to be linked to the creation of secondary reductants prior to the reduction of pollutants and the removal of the layer of iron oxide [17].

### 3.5. Regeneration and Re-Use of Columns

To attain the recycling performance of adsorbent, the performance of a fixed bed column was investigated to repeat the experiment of the adsorption-desorption cycle. Desorption was achieved by passing a 0.1 mol·L^−1^ NaOH solution to the bed in the upward direction of flow at a rate of 8 mL·min^−1^, which was slightly less as compared to sorption flow rate (10 mL·min^−1^), resulting in a smaller volume of regenerant and a higher concentration, allowing for cost-effective metal ion recovery. With the help of 800 mL distilled water, the desorbed column was then washed once again at a flow rate of 25 mL·min^−1^ in an upwards flow direction.

With a 20 cm bed depth, 1.0 mg·L^−1^ metal ion, and 10 mL·min^−1^ flow rate, the column tests for nZNI/AC were carried out. In comparison to the first cycle, the adsorption capacity had decreased after NaOH washing. However, without WMF, the adsorption capacity decreased by 13.8% and 22.7% in the second, and by 37.6% and 52.12% in the third cycle. The major reason is that as WMF promotes corrosion, Fe^2+^ flows out of the fixed bed, causing a decrease in adsorption sites and, and as a result, the fixed bed removes Sb(III).

### 3.6. Mechanism of Sb(III) Removal

Sb(III) oxidation by O_2_ is extremely sluggish [18,20]. However, it has been claimed that when ZVI is corroded by oxygen, powerful oxidants are produced that can oxidize a variety of organic and inorganic molecules. Two electrons are transported from Fe^0^ surfaces to oxygen (reaction (3)) under acidic conditions, resulting in hydrogen peroxide (H_2_O_2_). At low pH, the interaction of H_2_O_2_ with Fe^2+^ forms hydroxyl radicals (OH^−^), whereas at pH values over 5.0, it generates Fe(IV) via reaction (4–5) [21,22,23].

Our previous research found the activated carbon has the greatest impact on the oxidation of As(III) [8]. This is because the surface of activated carbon is rich in functional groups, which are oxidized when As(III) diffuses and adsorbs on the surface of activated carbon. Furthermore, because Sb(III) oxidizes more rapidly than As(III), Sb(III) can be quickly oxidized by AC [24]. According to the literature, Sb(III) can be readily oxidized by H_2_O_2_ in aqueous solutions [18,25], and Sb(III) oxidation can be catalyzed in both phases (aqueous and solid) on the mineral surfaces, particularly iron (hydr)oxides [26,27].

In the absence of WMF, the ZVI-mediated removal of Sb(III) generally consists of two timeframes. Initially, Sb(V) accumulation in the aqueous phase decreased the Sb(III) and Sb_tot_ concentrations, while the Sb_tot_ proportion rapidly decreased in the second period compared to the initial one, and this is attended by a decrease in the proportion of Sb(V), implying that the transformation of Sb limits the elimination of Sb(III) by ZVI. If no WMF exists, the absence of the two-period phenomenon in the change of Sb_tot_ with time during the ZVI mediated removal of Sb(III) at pH5.0 should be attributed to the rapid conversion of Sb(III) to Sb(V) in this case. Second, the use of WMF significantly enhanced the rate of removal of Sb(III) by ZVI at various pH levels. This is because of the accelerated ZVI corrosion and Sb(III) to Sb(V) oxidation in the presence of WMF.

**Figure 6:**
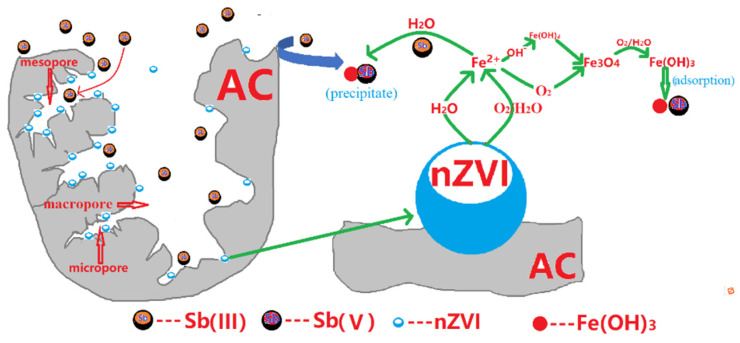
Illustration of the major reactions occurring in the nZVI/AC-H_2_O-O_2_ system and oxidation and sequestration mechanisms of Sb(III).

Based on the above results and discussion, the removal of Sb(III) was a complex process that included surface adsorption and oxidation. Figure 6 depicts the mechanism of Sb(III) removal. The elimination of Sb(III) was primarily accomplished through three processes: (1) most of the Sb(III) was adsorbed on the nZVI/AC surface (Equation (6)); (2) electrons were transferred from Sb(III) to the generated Fe(III) oxyhydroxide and AC (Equations (7) and (8)) in solution, resulting in a part of Sb(III) being rapidly oxidized to Sb(V) (Equation (9)) [7]; and (3) the resulting Sb(V) was adsorbed further on the nZVI/AC surface (Equation (10)) [28]. Sb(III) and Sb(V) are barely detectable in the supernatants after the reaction, implying that Sb(III) and produced Sb(V) were bound to the nZVI/AC surface.

Fe^0^ + O_2_ + 2H → Fe^2+^ + H_2_O_2_ (3)

Fe^2+^ + H_2_O_2_ → Fe^3^^+^ + ·OH + OH^−^ (4)

Fe^2+^ + H_2_O_2_ → Fe(IV) + H_2_O (5)

2≡Fe(OH) + Sb(OH)_3_ → (≡FeO)_2_Sb(OH) + 2H_2_O (6)

2Fe^0^ + O_2_ + 2H_2_O → 2Fe^2+^ + 4OH^−^ (7)

4Fe(OH)_2_ + O_2_ + 2H_2_O → 4Fe(OH)_3_ (8)

2Fe(OH)_3_ + Sb(OH)_3_ → 2Fe(OH)_2_ + H_3_SbO_4_ + H_2_O (9)

(10) $$2\equiv \mathrm{Fe}(\mathrm{OH})+{\mathrm{Sb}(\mathrm{OH})}_{6}^{-}\to (\equiv \mathrm{FeO}{)}_{2}{\mathrm{Sb}(\mathrm{OH})}_{4}^{-}+2H_{2}O$$

## 4. Conclusions

The study described the enhanced sequestration of Sb (III) by nZVI/AC under the influence of WMF at pH 5.0–9.0. The use of WMF improved the disappearance rate constants of Sb (III) over the pH range of 5.0–9.0 by nZVI/AC. The increased corrosion of ZVI was the cause of the WMF-induced increase sequestration and oxidation of Sb(III). The fix-bed column magnetic reactor outperformed its non-magnetic equivalent in terms of Sb(III) removal by nZVI/AC, indicating that WMF might be used to improve the ZVI-based Sb(III) removal in practical applications.

## Figures and Tables

**Table 1:** Main features of nZVI/AC.

Thickness	Shape	Diameter	Fe Content	Total Pore Volume	BET Surface Area
~20 nm	flakes	<100 nm	~8.2%	0.45 cm^3^/g	821.7 m^2^/g
